# A Cross-Sectional Study: What Contributes to Nursing Students’ Clinical Reasoning Competence?

**DOI:** 10.3390/ijerph18136833

**Published:** 2021-06-25

**Authors:** Soomin Hong, JuHee Lee, Yeonsoo Jang, Yoonju Lee

**Affiliations:** 1 College of Nursing, Yonsei University, Seoul 03722, Korea; soominsnow@gmail.com; 2 Mo-Im Kim Nursing Research Institute, College of Nursing, Yonsei University, Seoul 03722, Korea; ysjang517@yuhs.ac; 3 Research Institute of Nursing Science, College of Nursing, Pusan National University, Busan 50612, Korea; lyj@pusan.ac.kr

**Keywords:** students, nursing, clinical reasoning, problem solving, self-efficacy, clinical practicum, nursing education research

## Abstract

Clinical reasoning is a vital competence for nursing students, as it is required for solving problems arising in complex clinical situations. Identifying the factors that influence nursing students’ clinical reasoning competence in the social context can help their implicit educational needs. Therefore, this study aimed to determine the factors associated with developing clinical reasoning competency among undergraduate nursing students. In total, 206 senior nursing students were included in this study. Self-reported measures were used to obtain data on participants’ clinical reasoning competence, problem-solving abilities, academic self-efficacy, and level of clinical practicum stress. Relationships among continuous variables were analyzed using Pearson’s correlation coefficients. A multiple linear regression analysis was conducted to identify factors related to clinical reasoning competence. Our findings show that participants with better problem-solving abilities and academic self-efficacy perceived themselves as having higher levels of clinical reasoning competence. Nursing students with lower clinical practicum stress reported higher clinical reasoning competence. Significant factors identified were younger age and subcategories of problem-solving ability such as problem clarification, alternative solution development, planning/implementation, and self-regulated efficacy. Our findings highlight essential factors necessary for developing a nursing curriculum that contributes to professional nurses’ clinical reasoning competence.

## 1. Introduction

### 1.1. Background and Purpose of the Study

Clinical reasoning enhances nursing students’ problem-solving abilities in increasingly complex clinical situations. Clinical reasoning competence is regarded as a unique and dynamic process facilitating in-depth analyses of patients’ health problems, enabling safe nursing care [1]. Therefore, clinical reasoning is emphasized in nursing education. For example, in South Korea, the Korean Accreditation Board of Nursing Education highlighted nursing students’ clinical reasoning abilities as being important in the process of measuring learning outcomes [2].

The nursing process is a reflective cycle involving interactions between nurses, their patients, and various environmental factors [3]. Clinical reasoning is neither temporary nor linear but a cyclical nursing process within the limits of patients’ circumstances and nurses’ knowledge or experience [4,5]. As nurses with clinical reasoning competence can provide timely person-centered care, which is crucial for patient safety, it is necessary for them to possess clinical reasoning competencies before entering the clinical field [6,7]. Nursing education focused on clinical reasoning competence can improve the ability to cope with complex and unstable situations when dealing with patients [8].

More evidence is required to create a rigorous curriculum for nursing students, although nurse educators have been making an effort to include systematic clinical reasoning competence [9,10]. There is a lack of research on the factors associated with nursing students’ clinical reasoning competence. To develop a curriculum that facilitates the development of clinical reasoning competence, these factors must be identified in the social and educational context. Therefore, this study aimed to determine and analyze the factors associated with clinical reasoning competence in undergraduate nursing students.

### 1.2. Literature Review

The concept related to clinical reasoning has been emphasized in nursing education and practice [10]. Clinical reasoning, critical thinking, and clinical judgment are often used as a synonym in nursing studies. Critical thinking is a kind of supportive thinking for clinical reasoning, while clinical judgment results from the clinical reasoning process [1,8]. Since the early 2000s, researchers have proposed several theoretical models regarding clinical reasoning and its development, which are fundamental to nursing education [5,11,12,13,14]. First, Pesut and Herman (1999) proposed the outcome-present state-test (OPT) model of clinical reasoning, which addresses the process of dealing with information cyclically and simultaneously [14]. The key to the OPT model is the continuous reflection on one’s clinical reasoning, the presentation of exact results, and patient-related decisions and judgments that are made by identifying the personalized nursing care needs of the patients [8]. Clinical reasoning is the process of considering possible preventive methods and management of patients’ problems; the decisions made through this process are called clinical judgment [1]. Next, Tanner (2006) presented the clinical judgment model (CJM) by which registered nurses solve complex and uncertain patient-related problems [5]. By applying the CJM, nursing students can make clinical judgments in complex clinical situations using theoretical knowledge and clinical reasoning [5]. Based on the CJM, the Lasater Clinical Judgment Rubric (LCJR), which focuses on clinical judgments, was proposed [13]. Lasater and Nielson (2009) implemented reflective journaling, based on Tanner’s CJM and Lasater’s LCJR, in the curriculum for nursing students to promote clinical reasoning competence [15]. In their study, nursing students were asked to maintain a reflective journal after high-fidelity simulations. They found that reflective journaling improved clinical judgment and reasoning capabilities through self-reflection and instructor feedback [15]. Based on the findings of their study, Lasater and Nielson (2009) emphasized that a systematic mode of educational intervention, which is built on academic theories, is required to provide nursing students with the necessary education [15]. According to Simmons’ (2010) concept analysis of clinical reasoning [16], core attributes—such as *intuition*, *metacognition*, and *inference*—were linked to patient assessment and data organization concepts when solving patients’ health problems. Recent educational programs have aimed to enhance clinical reasoning competence by using diverse strategies, such as simulation-based [17,18], case- and problem-based [19,20], and mobile device-based learning [21].

Problem-solving is the ability to address patients’ needs by systematically analyzing the given information. Although it is not innate, it can be acquired through organized education and training [22,23]. Problem-solving ability has been an essential variable in evaluating nursing educational intervention [24,25]. Nursing students with strong problem-solving abilities had high levels of patient safety skills, communication ability, and clinical proficiency [26]. However, problem-solving ability among nursing students differs significantly according to their interactions and interpersonal functioning [27]. Recent studies highlight that problem-solving ability can find appropriate answers in difficult and complex clinical situations. In this study, problem-solving ability was considered a factor that influences nursing students’ clinical reasoning competence.

Self-efficacy is the confidence that enables students to overcome challenging situations [28]. Among the different types of self-efficacy, academic self-efficacy is motivated by the belief that one’s ability can be shown in an educational setting. Academic self-efficacy is regarded as learners’ ability to manipulate proper knowledge and performance through their judgments in academic situations [29]. Learning situations that encourage nursing students to improve their clinical reasoning ability are challenging. However, academic self-efficacy promotes active involvement in experiential teaching methods that can increase clinical reasoning competence [30]. Additionally, academic self-efficacy contributes to “*self-instructed performances*” [31] such as goal setting, effort, and perseverance to endure difficulties with a perceptual belief [32,33]. In this study, academic self-efficacy was an independent factor that is essential for reflection and motivation of nursing students.

Improving the clinical reasoning competence of students has been required through actual clinical nursing experiences [34]. However, few previous studies have explored the relationship between clinical practicums and clinical reasoning. Classroom-acquired knowledge is applied during clinical practicums and developed by working with actual patients [35]. Nursing students can gain metacognition skills, which are vital for clinical reasoning, during this circular process [8,35]. In clinical practicums, students experience actual nursing situations that provide opportunities to think like a professional nurse and prepare to become one [36,37]. However, during this process, nursing students encounter stressful clinical situations that reduce their interest in nursing [38]. This stress can undermine their clinical reasoning competence [37,39]. Therefore, nursing students who often experience stressful situations during their clinical practicum are less motivated and have fewer opportunities to improve their clinical reasoning competence.

Identifying factors associated with nursing students’ clinical reasoning competence will help define students’ implicit curricular needs. Furthermore, applying teaching and learning methods that consider these factors will ultimately increase nursing students’ clinical reasoning competence.

## 2. Materials and Methods

### 2.1. Study Design

This study applied a cross-sectional survey design. The study is reported in accordance with the STROBE guidelines [40].

### 2.2. Sample

Data were obtained from 223 undergraduate senior nursing students from four nursing schools in South Korea from March to May 2018. Using G*power 3.1.9.2 (Heinrich-Heine-Universität Düsseldorf, Düsseldorf, Germany), the sample size required to detect significant differences was calculated to be 187, with a significance level of type I error (α) of 0.05, power (1-β) of 0.9, an effect size 0.15, and 19 predictors. Considering a response rate of 85%, the targeted number of participants was 223. By the convenience sampling method at four nursing schools, 223 students were recruited. Of which 206 students (92.4%) who completed the survey were included in this study; this met the 187-student requirement. All the participants were able to self-report in Korean.

### 2.3. Ethical Considerations and Data Collection

Ethical approval was obtained from the Institutional Review Board of the relevant university in Seoul, South Korea (Y-2018-0002). Data were collected after receiving signed informed consent forms from the participants. Participants were informed they could ask any questions and leave the study at any time.

### 2.4. Measures

Relevant permissions were obtained to use all the study instruments. General characteristics such as gender, age, satisfaction with the nursing major, and grade point average were collected using a self-report questionnaire.

Clinical reasoning competence was evaluated using the Nurses Clinical Reasoning Scale (NCRS) developed by Liou et al. [41], which was translated and validated for use in the Korean context by Joung and Han [42]. We confirmed the scale’s validity in the context of undergraduate nursing students with its developer. The NCRS comprises 15 items scored on a 5-point Likert-type scale ranging from 1 (strongly disagree) to 5 (strongly agree). Higher scores indicate higher clinical reasoning competence. Cronbach’s alpha was 0.94 in the original study [41], 0.93 in the Korean translation and validation study [42], and 0.85 in the present study.

Problem-solving ability was measured using the Problem-Solving Ability Scale developed by the Korean Educational Development Institute [43]. This scale consists of 45 items across five subscales. Items on each subscale are scored on a 5-point Likert-type scale ranging from 1 (very rarely) to 5 (very often). Higher scores indicate greater problem-solving ability. Cronbach’s alpha was 0.87 in the present study.

Academic self-efficacy was evaluated using the Academic Self-Efficacy Scale developed by Kim and Park [29], which comprises 28 items across three subscales: task difficulty, self-regulated efficacy, and confidence. Items on each subscale are scored on a 6-point Likert-type scale ranging from 1 (strongly disagree) to 6 (strongly agree). Higher scores indicate better academic self-efficacy. In this study, Cronbach’s alpha was 0.88.

Participants answered 20 of the 59 items in the Stress Scale for Korean Nursing [44]. The 20 questions addressed stress stemming from a clinical practicum. The scale developers approved the use of these 20 items for the present study. The tool is scored on a 5-point Likert-type scale ranging from 1 (never) to 5 (almost always). Higher scores indicate higher levels of clinical practicum-related stress. Cronbach’s alpha was 0.89 in the present study.

### 2.5. Statistical Analysis

Data were analyzed using SPSS version 23.0 (IBM, Armonk, NY, USA). After a normality test, the means and standard deviations (SD) of the participants’ general characteristics were analyzed to determine clinical reasoning competence and other variables. Relationships between the continuous variables were identified using Pearson’s correlation coefficients. Multiple linear regression analyses were performed to investigate factors related to clinical reasoning competence.

## 3. Results

### 3.1. Participants’ Characteristics

In total, 206 participants were included; their characteristics are described in Table 1. Most participants were female (86.4%), and their mean age was 22.38 years (SD = 1.68). The majority were satisfied with their nursing major (63.6%). The mean score for clinical reasoning competence was 50.90 (SD = 6.46). The means for problem-solving ability and academic self-efficacy were 162.71 (SD = 13.66) and 105.44 (SD = 16.02), respectively. The mean clinical practicum stress score was 53.14 (SD = 13.79).

### 3.2. Correlations between Clinical Reasoning Competence and Other Factors

Table 2 shows the correlations between clinical reasoning competence, problem-solving ability, academic self-efficacy, and clinical practicum-related stress. Participants with higher problem-solving ability (*r* = 0.334, *p* < 0.001), higher academic self-efficacy (*r* = 0.303, *p* < 0.001), and lower clinical practicum-related stress (*r* = −0.141, *p* < 0.043) were perceived as having better clinical reasoning competence.

### 3.3. Factors Associated with Clinical Reasoning Competence

For the multiple regression analysis, clinical reasoning competence was the dependent variable, and the independent variables were the general characteristics, problem-solving ability, academic self-efficacy, and clinical practicum-related stress (Table 3). The multicollinearity was reviewed by identifying the variance inflation factor (VIF), which ranged from 1.100 to 3.427. All independent variables were selected according to the VIF. The normal probability plot (Q–Q plot) was reviewed to verify the normality, and the residual plot was used to judge homoscedasticity. This study satisfied the fundamental assumptions of the regression model. In addition, the Durbin–Watson value was 2.017, which is close to the reference value 2 and was not close to 0 or 4. It was deemed that there was no correlation between residuals, making the multiple regression model suitable [45]. As presented in Table 3, each variable subcategory explained 20.3% of the variance in clinical reasoning (adjusted *R*^2^ = 0.203, *p* < 0.001). Among the subcategories, age under 21 years (β = 3.930, *p* = 0.013), problem clarification (β = 0.597, *p* = 0.003), alternative solution development (β = −0.351, *p* = 0.012), planning and implementation as problem-solving abilities (β = 0.247, *p* = 0.022), and self-regulated efficacy in relation to academic self-efficacy (β = 0.162, *p* = 0.039) were found to be significant. Regarding general characteristics, younger students showed better clinical reasoning competency. Among the subcategories of problem-solving ability, problem clarification as well as planning and implementation were identified as factors that could potentially enhance clinical reasoning competence. However, participants with high alternative solution development scores displayed low clinical reasoning competence. In academic self-efficacy, self-regulated efficacy was associated with clinical reasoning competence.

## 4. Discussion

This study was conducted to identify the factors associated with nursing students’ development of clinical reasoning competency. The following findings explain additional factors that are related to clinical reasoning.

There was a positive relationship between problem-solving ability and clinical reasoning competence, which is consistent with previous studies [19,46]. Problem-solving ability means that one is able to successfully apply their knowledge to resolve a problematic situation [47]. According to Simmons, nursing care is “*deliberate*”, and nurses need to be “*intuitional*” and “*heuristic*” when addressing patient-related problems [16,48]. These aforementioned attributes of clinical reasoning competence can influence one’s problem-solving ability. Nurses who have clinical reasoning competence understand the situation and successfully resolve problems in complex and diverse clinical environments [5]. Clinical reasoning skills weave information like a web and integrate data contextually. Thereby, nurses perceive patients’ data as individualized information when solving problems [7,8,11]. A study found that nurses who had problem-solving competence that relied on clinical reasoning were able to meet patients’ needs and expectations [48]. In this study, nurses who care for cancer patients needed clinical reasoning to cope with cancer patients’ complex problems while providing high-quality and safe nursing care [48].

Nursing students’ academic self-efficacy in this study was significantly associated with clinical reasoning competence. Of the three domains of academic self-efficacy in this study, self-regulated efficacy was found to affect clinical reasoning competence. Nursing education, which included nursing practices informed by the OPT model for clinical reasoning, led to self-efficacy development [49]. Education that includes both evidence-based and individualized nursing care is vital for providing comprehensive nursing care using clinical reasoning. That means “*knowledge work*” is required to interpret data and apply evidence-based nursing [8]. Constructivist educational tactics such as case- or problem-based learning are needed to understand nursing in a global context [24,50]. These aspects of nursing education require self-regulated learning steps that consider the learner’s self-efficacy. Self-regulated efficacy is crucial in inclusive and multidimensional education settings [25,51]. Henderson et al. (2018) developed the check-in and check-out (CICO) curriculum for the continual enhancement of nursing students’ academic self-efficacy [52]. The CICO curriculum includes self-regulated learning goals and continuous interactions between the teachers and students. Self-efficacy is considered vital for nursing students’ routine education to be effective [53].

In addition, self-efficacy is especially important in new and challenging situations. Educational methods and strategies have been changing recently due to the coronavirus disease 2019 (COVID-19) pandemic. As socially-distanced learning has been employed by many colleges since the COVID-19 pandemic, there is an increased emphasis on nursing students having acceptable self-directed ability [54]. For example, medical and nursing educators have applied learning methods to improve clinical proficiency during the pandemic [55,56]. Since direct interactions with patients during a pandemic increase nursing students’ exposure to the disease, educators provided simulated practicum classes, group activities, and problem-based learning online [57,58]. These online classes, which are presented in flexible formats, strengthen students’ judgment and clinical reasoning in a safe environment. It is predicted that this sudden change in education will have a significant impact on education worldwide, even after this pandemic ends [55]. A previous study found that enhanced self-regulation or self-efficacy could boost the efficacy of online learning [59]. We need to prepare the changeable educational stream to improve students’ self-efficacy and clinical reasoning.

Nursing students improve their competencies through a comprehensive nursing curriculum, not through a particular moment [60]. In this “becoming a nurse” process, integrating nursing knowledge and practice was connected to using clinical reasoning [60]. Yamadala’s (2016) study developed a geriatric medicine curriculum that included problem-based learning, through which the medical students encountered real examples that enabled them to learn in-depth about solving problems related to elderly patients [61,62]. Another study, conducted on pharmacology students found that their problem-solving ability improved after participating in the Community Parametric Program in which they faced actual patients [62]. Similarly, advanced educational strategies and tools can help improve nursing students’ care provision from novice to expert [60]. For example, a teaching method that utilizes real cases could be effective in improving nursing students’ problem-solving ability. Long-term strategies are needed to promote nursing students’ clinical reasoning competence. Organized teaching and learning methods can help close the gap between theory and practice for nursing students by improving their clinical reasoning. A previous systematic review reported that problem-based learning could improve nurses’ integrative thinking and reflection processes by providing comprehensive clinical situations [50]. Additionally, nursing curriculums that included simulations and provided realistic situations helped improve students’ nursing procedures, communication skills, empathy, clinical judgment, and self-efficacy [63,64].

This study had several limitations. First, while clinical practicums and curriculums differ based on the school, these differences were not considered during the analyses in this study. Therefore, the findings related to clinical practicums and educational experiences have limited generalizability. Second, most participants did their clinical practicum in general hospitals; however, their clinical practicum stress scores were widely distributed. Thus, clinical practicum-related stress was not included in the study’s clinical reasoning competence regression model. Finally, while we identified several factors associated with clinical reasoning competence, the cross-sectional design limited causal inferences; thus, future studies should employ longitudinal designs to determine the relevant factors definitively.

Despite such limitations, this study is significant as it measured nursing students’ clinical reasoning competence and uncovered related factors. Our findings can be used to develop effective nursing education strategies and tactics for improving clinical reasoning competence. Furthermore, the present results can contribute to developing research-based educational programs. Educational programs that enhance clinical reasoning support nursing students and help bridge the gap between education and practice.

## 5. Conclusions

Clinical reasoning competence plays a pivotal role in solving various problems that occur in clinical nursing settings. This study found that problem-solving ability and academic self-efficacy were significant factors that affect clinical reasoning competence. Nursing education requires long-term perspectives and strategies for promoting nursing students’ clinical reasoning competence. Teaching and learning methods that enhance problem-solving ability in clinical situations and ways that improve self-efficacy are required in nursing education. While this study provided valuable data for enhancing nursing students’ clinical reasoning competence, further research is needed. Future studies such as cross-cultural or longitudinal studies are required to identify the specific factors that influence clinical reasoning competence.

## Figures and Tables

**Table 1:** Participants’ characteristics in this study (*N* = 206).

Categories	*n*	(%)	No of Questions	Scale	Min.	Max	Mean (SD)
Gender							
Male	28	13.6					
Female	178	86.4					
Age (years)							22.38 (1.68)
≤21	70	34.0					
22–23	98	47.6					
≥24	38	18.4					
Satisfaction with nursing major							
Satisfied	131	63.6					
Moderate	44	21.4					
Dissatisfied	31	15.0					
Grade point average (GPA)							
2.0–2.9	21	10.2					
3.0–3.9	149	72.3					
≥4.0	36	17.5					
Clinical reasoning competence			15	1–5	32	68	50.90 (0.79)
Problem-solving ability			45	1–5	116	199	162.71 (13.66)
Academic self- efficacy			28	1–6	63	150	105.44 (16.02)
Clinical practicum stress			20	1–5	22	97	53.41 (13.79)

**Table 2:** Correlation between clinical reasoning competence, problem-solving ability, academic self-efficacy, and clinical practicum stress (*N* = 206).

Variables	Clinical Reasoning Competence	Problem-Solving Ability	Academic Self-Efficacy	Clinical Practicum Stress
	*r* (*p*)	*r* (*p*)	*r* (*p*)	*r* (*p*)
Clinical reasoning competence	1			
Problem-solving ability (Total)	0.334 (<0.001) **	1		
Problem clarification	0.271 (<0.001) **			
Causal analysis	0.243 (<0.001) **			
Alternative solution generation	0.123 (0.077)			
Planning/imple-mentation	0.292 (<0.001) **			
Performanceassessment	0.235 (0.001) **			
Academic self-efficacy (Total)	0.303 (<0.001) **	0.420 (<0.001) **	1	
Task difficulty	0.168 (0.016) *	0.322 (<0.001) **		
Self-regulated efficacy	0.324 (<0.001) **	0.477 (<0.001) **		
Confidence	0.170 (0.014) *	0.118 (0.092)		
Clinical practicum stress (Total)	−0.141 (0.043) *	0.020 (0.775)	−0.361 (<0.001) **	1
Instructor and healthcare team	−0.114 (0.103)	0.043 (0.538)	−0.381 (<0.001) **	
Client	−0.105 (0.135)	−0.034 (0.623)	−0.276 (<0.001) **	
Clinical environment	−0.102 (0.145)	0.025 (0.727)	−0.133 (0.056)	
Student	−0.124 (0.075)	0.072 (0.305)	−0.327 (<0.001) **	

* *p* < 0.05, ** *p* < 0.01.

**Table 3:** Factors influencing clinical reasoning competence (*N* = 206).

Variables	β	S.E.	*t*	*p*	95% CI	
					Lower	Upper
Characteristics						
Gender (Male) ^a^	2.736	1.613	1.697	0.091	−0.446	5.918
Age ^b^ (years)						
≤21	3.930	1.570	2.504	0.013 *	0.834	7.027
22–23	2.289	1.470	1.557	0.121	−0.612	5.189
Satisfaction with nursing major ^c^						
Moderate	−0.264	1.028	−0.257	0.797	−2.292	1.763
Dissatisfied	2.040	1.251	1.631	0.105	−0.428	4.508
Grade point average (GPA) ^d^						
3.0–3.9	1.540	1.424	1.081	0.281	−1.270	4.350
≥4.0	0.141	1.764	0.080	0.937	−3.339	3.620
Problem-solving ability						
Problem clarification	0.597	0.200	0.2991	0.003 **	0.203	0.991
Causal analysis	0.253	0.135	1.870	0.063	−0.014	0.519
Alternative solution development	−0.351	0.138	−2.540	0.012 *	−0.624	−0.078
Planning/implementation	0.247	0.107	2.317	0.022 *	0.037	0.457
Performance assessment	0.159	0.110	1.448	0.149	−0.058	0.375
Academic self-efficacy						
Task difficulty	0.045	0.062	0.729	0.467	−0.077	0.167
Self-regulated efficacy	0.162	0.078	2.081	0.039 *	0.008	0.316
Confidence	−0.002	0.067	−0.023	0.982	−0.133	0.130
Clinical practicum stress						
Instructor and healthcare team	−0.169	0.123	−1.368	0.173	−0.412	0.075
Client	0.026	0.079	0.327	0.744	−0.130	0.181
Clinical environment	0.035	0.137	0.256	0.798	−0.235	0.306
Student	−0.286	0.181	−1.574	0.117	−0.644	0.072
*R* = 0.526, *R*^2^ = 0.277, Adjusted *R*^2^ = 0.203						
*F* = 3.744, *p* < 0.001						
Durbin–Watson = 2.017						

* *p* < 0.05, ** *p* < 0.01, dummy code reference (^a^: Female, ^b^: ≥24, ^c^: Satisfied, ^d^: 2.0–2.9).

## Data Availability

The data presented in this study are available on request from the corresponding author. The data are not publicly available due to sensitive information.
